# The impact of insecticide treated curtains on dengue virus transmission: A cluster randomized trial in Iquitos, Peru

**DOI:** 10.1371/journal.pntd.0008097

**Published:** 2020-04-10

**Authors:** Audrey Lenhart, Amy C. Morrison, Valerie A. Paz-Soldan, Brett M. Forshey, Jhonny J. Cordova-Lopez, Helvio Astete, John P. Elder, Moises Sihuincha, Esther E. Gotlieb, Eric S. Halsey, Tadeusz J. Kochel, Thomas W. Scott, Neal Alexander, Philip J. McCall

**Affiliations:** 1 Vector Biology Department, Liverpool School of Tropical Medicine, Liverpool, United Kingdom; 2 Department of Pathology, Microbiology, and Immunology, School of Veterinary Medicine, University of California, Davis, California, United States of America; 3 Department of Virology, U.S. Naval Medical Research Unit-6, Lima and Iquitos, Peru; 4 Department of Global Community Health and Behavioral Sciences, Tulane School of Public Health and Tropical Medicine, New Orleans, Louisiana, United States of America; 5 Facultad de Salud Pública y Administración, Universidad Peruana Cayetano Heredia, Lima, Peru; 6 San Diego State University, San Diego, California, United States of America; 7 Director, Department of Internal Medicine, Hospital de Apoyo Iquitos, Peru; 8 Department of Entomology and Nematology, University of California, Davis, California, United States of America; 9 MRC Tropical Epidemiology Group, London School of Hygiene & Tropical Medicine, London, United Kingdom; Faculty of Science, Mahidol University, THAILAND

## Abstract

Dengue is one of the most important vector-borne diseases, resulting in an estimated hundreds of millions of infections annually throughout the tropics. Control of dengue is heavily dependent upon control of its primary mosquito vector, *Aedes aegypti*. Innovative interventions that are effective at targeting the adult stage of the mosquito are needed to increase the options for effective control. The use of insecticide-treated curtains (ITCs) has previously been shown to significantly reduce the abundance of *Ae*. *aegypti* in and around homes, but the impact of ITCs on dengue virus (DENV) transmission has not been rigorously quantified. A parallel arm cluster-randomized controlled trial was conducted in Iquitos, Peru to quantify the impact of ITCs on DENV seroconversion as measured through plaque-reduction neutralization tests. Seroconversion data showed that individuals living in the clusters that received ITCs were at greater risk to seroconverting to DENV, with an average seroconversion rate of 50.6 per 100 person-years (PY) (CI: 29.9–71.9), while those in the control arm had an average seroconversion rate of 37.4 per 100 PY (CI: 15.2–51.7). ITCs lost their insecticidal efficacy within 6 months of deployment, necessitating re-treatment with insecticide. Entomological indicators did not show statistically significant differences between ITC and non-ITC clusters. It’s unclear how the lack of protective efficacy reported here is attributable to simple failure of the intervention to protect against *Ae*. *aegypti* bites, or the presence of a faulty intervention during much of the follow-up period. The higher risk of dengue seroconversion that was detected in the ITC clusters may have arisen due to a false sense of security that inadvertently led to less routine protective behaviors on the part of households that received the ITCs. Our study provides important lessons learned for conducting cluster randomized trials for vector control interventions against *Aedes*-transmitted virus infections.

## Author summary

Dengue is one of the most important mosquito-borne diseases affecting humans, resulting in an estimated hundreds of millions of infections annually throughout the tropics. To control dengue, most public health programs use a variety of methods to kill the primary mosquito vector, *Aedes aegypti*. Water holding containers that harbor larvae (and other immature stages) are treated or eliminated. During emergencies, large insecticide spray campaigns are deployed to kill infected adult mosquitoes. Innovative interventions that are effective at targeting adult mosquitoes in sustainable ways are needed to increase the options for control of dengue and other *Aedes* borne virus diseases. The use of insecticide-treated curtains (ITCs) has previously been shown to significantly reduce *Ae*. *aegypti* numbers in and around homes, but the impact of ITCs on dengue virus (DENV) transmission has not previously been quantified. Using a rigorous study design in which 10 clusters (~90 houses per cluster) were provided multiple ITCs to place in their homes was compared to 10 clusters of homes without ITCs. Assignment of which clusters received ITCs was randomized. Blood samples were obtained at 9-month intervals from residents living in all the clusters, so that people with serological evidence of a DENV infection could be identified by comparing paired samples. Seroconversion data showed that individuals living in the clusters that received ITCs were at greater risk to DENV seroconverting, with an average seroconversion rate of 50.6 per 100 person-years (PY) (CI: 29.9–71.9). Conversely, those in the control arm had an average seroconversion rate of 37.4 per 100 PY (CI: 15.2–51.7). ITCs lost their insecticidal efficacy within 6 months of deployment, necessitating re-treatment with insecticide. *Ae*. *aegypti* populations did not show statistically significant differences between ITC and non-ITC clusters. The reason for higher transmission in the ITC treated clusters could be attributable to failure of the curtains (loss of efficacy) and/or that the curtains were not sufficiently effective at protecting against mosquito bites. The higher risk of DENV seroconversion in ITC clusters may be due to a false sense of security that inadvertently led to less routine protective behaviors on the part of households that received the ITC.

## Introduction

Dengue is a major public health problem, with an estimated 390 million dengue virus (DENV) infections occurring annually worldwide [1]. Control of the peridomestic DENV mosquito vector, *Aedes aegypti* (and to a lesser extent, *Aedes albopictus*), is currently the primary preventive measure. Existing vector control methods largely target immature mosquito stages, requiring continuous effort by communities [2], and are often challenging to sustain [3]. Because adult mosquitoes are responsible for virus transmission, targeting adults, rather than the aquatic stages, should have the most direct impact on virus transmission. The most common interventions targeting adult *Ae*. *aegypti* employ ultra-low volume (ULV) insecticide spray applications. ULV spraying does not offer any residual insecticidal effect, and studies indicate that ULV spraying is ineffective unless repeated frequently at closely timed intervals [4]. Hence, it is most practical when employed for outbreak response rather than for routine dengue control [4–6]. Novel interventions utilizing residual insecticides that target adult *Ae*. *aegypti* are needed to increase the options for effective dengue vector control programs.

Insecticide-treated materials (ITMs) deployed as bednets are highly effective in preventing transmission of malaria [7] and other nocturnally transmitted vector-borne diseases including Chagas disease [8], leishmaniasis [9], and lymphatic filariasis [10]. Control of dengue diurnal vectors using ITMs has similarly been demonstrated, mainly as insecticide treated curtains (ITCs) [11–16]. The residual formulations of insecticides used on ITCs allow for a potentially long-lasting effect, and ITCs are ‘user-friendly’, requiring little additional work or behavioral change by householders. They are also well accepted by communities [17], because their perceived efficacy is reinforced by the reduction in other biting insects, cockroaches, houseflies and other insect pests [11].

Despite a body of evidence reporting the entomological impact of ITCs on *Ae*. *aegypti*, little is known about their epidemiological impact on dengue or other arboviral infections. Although preliminary evidence suggested that ITCs could impact *Ae*. *aegypti* populations at a level that could reduce DENV transmission [11, 16], the epidemiological effect has not been rigorously evaluated. To address this gap, we carried out a cluster-randomized controlled trial of ITCs in Iquitos, Peru.

DENV transmission re-emerged in Iquitos in 1990 after a 30-year absence, and successive epidemics occurred with subsequent DENV serotype invasions periodically since then [18–25]. Routine *Ae*. *aegypti* control in Iquitos consisted of larviciding and health education activities utilizing billboards, radio, and TV messages focusing on preventive vector control activities (removal and management of potential and actual larval habitats) and recognition of dengue symptoms, especially early warning signs of severe disease. In response to increases in reported dengue cases or elevated *Ae*. *aegypti* indices, emergency measures, including ULV spraying and city-wide cleanup campaigns (collection of water-holding containers), were employed [19, 21, 22, 26–30]. The extensive longitudinal data on the dynamics of serotype-specific DENV transmission over many years in Iquitos was used to design a vector control trial with epidemiological endpoints [26, 27, 31]. Herein, we report the outcomes of an Iquitos ITC trial.

## Materials and methods

### Ethical approval

This study received approval from the Institutional Review Boards (IRBs) at the Liverpool School of Tropical Medicine, the Tulane School of Public Health and Tropical Medicine, the London School of Hygiene and Tropical Medicine, the University of California at Davis, and the U.S. Naval Medical Research Center Detachment (now the U.S. Naval Medical Research Unit-6) in Peru (S1 Protocol). The latter had interinstitutional IRB agreements with the Tulane School of Public Health and Tropical Medicine and the University of California at Davis. The Regional Health Authority (DIRESA), the local branch of the Peruvian Ministry of Health, also provided approval. The trial was registered with the International Standard Randomized Controlled Trial Register: ISRCTN08474420. Verbal consent was obtained for ITC deployment and entomological monitoring activities, as approved by all IRBs. Written consent was obtained for all blood draws from study participants (≥ 18 years of age) or a parent or guardian (if the participant was between 3–17 years of age). Assent was obtained for all participants < 18 years of age, with written documentation of assent for all children > 7 years of age.

### Study site and design

**Fig 1:**
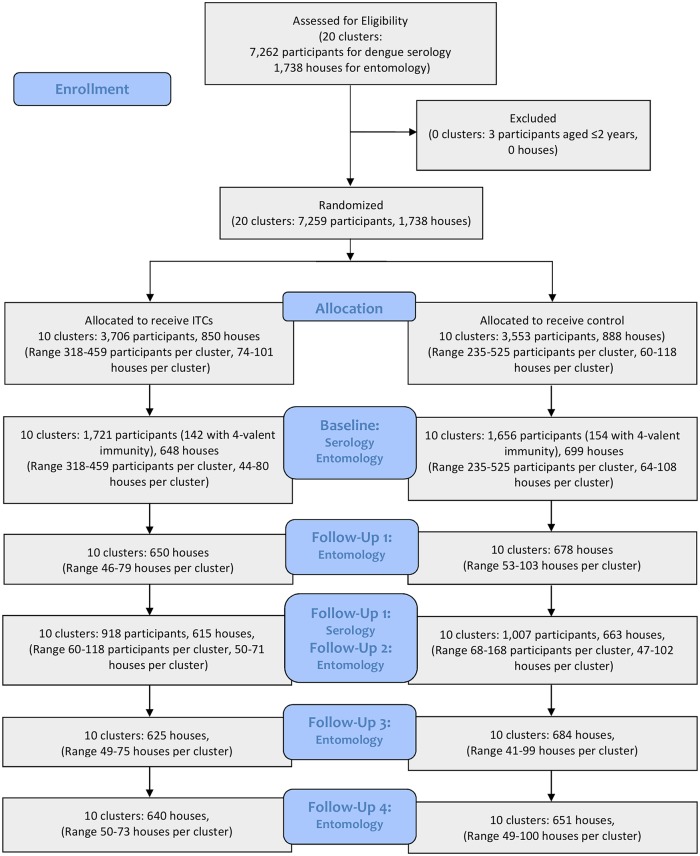
CONSORT flowchart describing the recruitment and retention of participants and allocation to each study arm.

**Fig 2:**
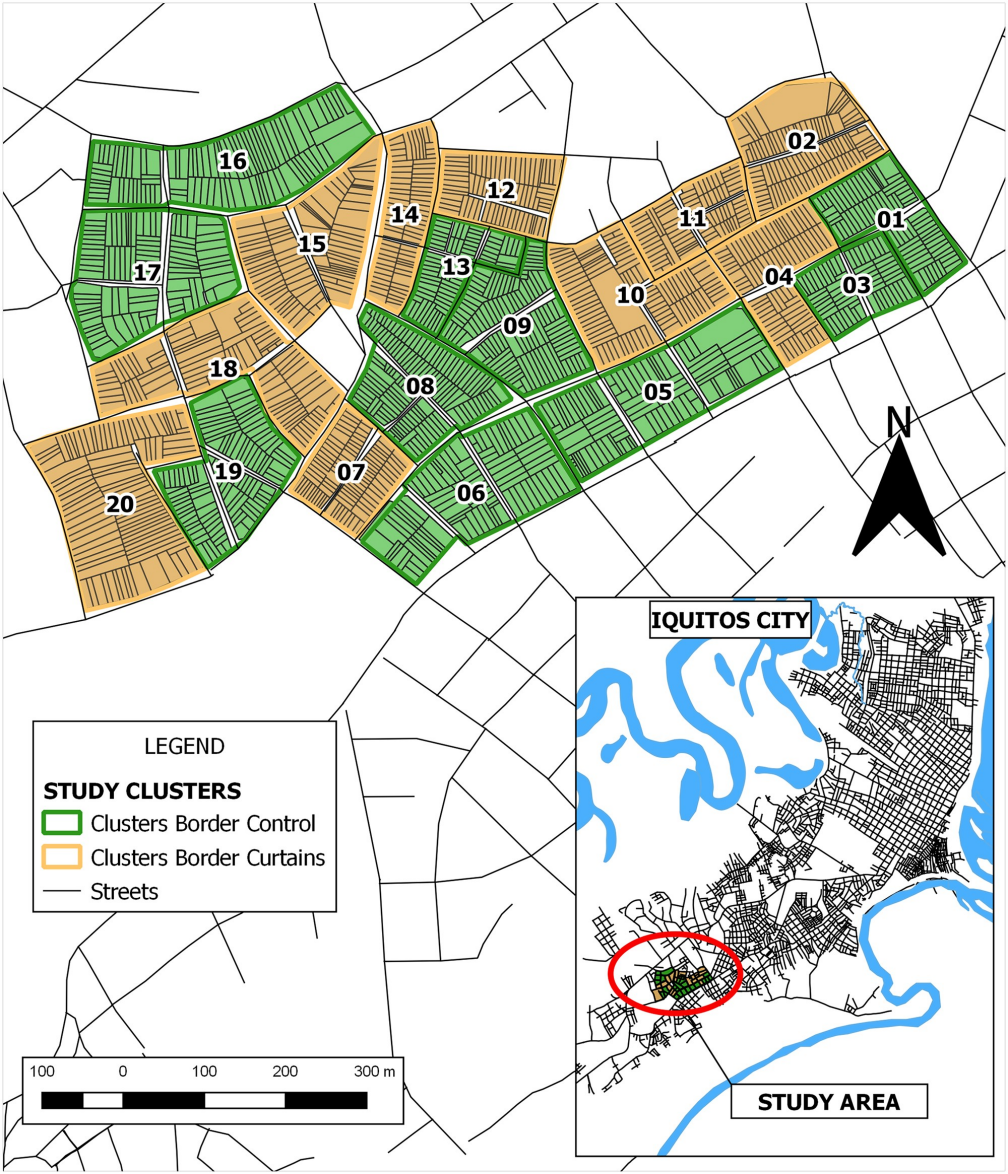
Map of the study area showing the location of the twenty clusters, randomized to either receive ITCs or act as an untreated control (no ITCs). This figure was created using QGIS software using shapefiles created as described previously [32, 33].

Our parallel arm cluster-randomized controlled trial began during October 2009 in the district of San Juan in Iquitos, which is located in the Amazon region of north-eastern Peru (73.2°W longitude, 3.7°S latitude, 120 m above sea level). The primary outcome measure was reduction of DENV seroconversion, as measured by detection of dengue-specific plaque reduction neutralizing antibodies in human blood taken from householders within the study area (Fig 1 and S1 Checklist). Twenty clusters (consisting of 1–3 city blocks each containing a minimum of 70 households) were selected for the study (Fig 2). In late September 2009, prior to commencement of field activities, treatment was randomized so that 10 clusters received ITCs and 10 clusters did not receive ITCs (control clusters). Clusters were allocated to the intervention or control arm by simple randomization using a lottery: each cluster was represented by a piece of paper which was drawn in turn from a bag by study personnel. ITCs were thus allocated at the start of the trial, which obviated the need for allocation concealment. Clusters were geographically contiguous in the same region of the city.

The sample size calculation was based on data from 2 previous studies [29, 34] using DENV PRNT status at 9-month intervals in residents of Iquitos. Hayes & Bennett’s [35] sample size calculation method for binary data was used with the following parameters, which were chosen to lie within the range of values found in the previous studies: average of 120 people at risk per clusters, between-cluster coefficient of variation of 0.30, significance level 5% (two-sided), and seroconversion rate in control and intervention clusters of 0.25 and 0.1375/year respectively (55% efficacy). Using these parameters, 10 clusters per arm were needed for 90% power. This was estimated to provide 4,000 blood samples at baseline (2,000 per arm; more than the 1350 required to detect a difference) and an estimated 2,000 at each subsequent sample period, assuming that >50% of the population remained susceptible to ≥1 serotype. All individuals above the age of 3 years living in the study area who consented to provide baseline and follow-up blood samples were enrolled in a longitudinal cohort. Blood samples were collected from the study population at baseline and at 9-months after the ITCs were distributed. No clusters were lost to follow-up (Fig 2).

#### Intervention

During November 2009, ITCs were distributed in the clusters randomly allocated to receive ITCs. Control clusters did not receive ITCs. The trial, therefore, was not blinded. Residents could request as many curtains as they wanted and directed staff to where they should be hung. Most were hung in windows, doors, walls, and used as room dividers. Participants could choose among pink, light blue, and dark blue curtain colors. Surveys of curtain coverage were carried out in December 2010 and June 2011, and additional curtains were distributed subsequently according to need, with a total of 4,227 ITCs distributed over the course of the trial. The ITCs distributed at the beginning of the trial were made from Permanet 2.0 (Vestergaard Frandsen, Lausanne, Switzerland; deltamethrin-treated).

Routine monitoring of insecticidal efficacy using WHO cone bioassays [36] was implemented. At baseline, 12 new curtains were tested and showed 100% mortality using the local susceptible Bellavista-Nanay *Ae*. *aegypti* strain. After the ITCs had been hanging for 6 months (May 2010), a representative sample of 18 curtains was collected from randomly selected houses, according to a matrix of characteristics (6 of each colour, exposed to either sun or shade and washed 0, 1 or >1 times). Results were highly variable and ranged from 34%-100% mortality, with 8 of the curtains falling below the 80% mortality threshold, with no discernible pattern attributable to curtain color, sun exposure, or washing frequency. After hanging for 8 months (July 2010), a further 18 ITCs were selected for testing using the same methodology. Results showed further declines in bioefficacy, with 13 of the curtains falling below the 80% mortality threshold (range: 14%-100%). After hanging for 11 months (October 2010), the same process was repeated and all except for 1 curtain fell below the 80% mortality threshold (range: 32%-98%). Therefore, to ensure an effective intervention was present in the treated households for the remaining period of the study, curtains were re-treated with deltamethrin in the form of KO Tab 123 (Bayer) during November 2010, with a total of 3,886 curtains (91.9%) re-treated. Further cone bioassays to assess ITC efficacy were conducted 1 month following re-treatment (January 2011) and 9-months following re-treatment (August 2011) using the susceptible New Orleans *Ae*. *aegypti* strain.

#### DENV transmission

After receiving informed consent, blood samples were collected by either finger stick or venipuncture, the former usually being more acceptable, at baseline and at 9-months post-ITC distribution. Samples were analyzed for DENV neutralizing antibodies using a plaque-reduction neutralization test (PRNT) with a 70% reduction for the cut-off (PRNT_70_). PRNT_70_ were performed as described by Morrison et al. [29] for each DENV serotype (1–4) at the following serum dilutions: 1:40, 1:80, 1:160, and 1:640. Probit analysis was carried out to determine the estimated endpoint titers for each serotype. A serum sample was considered positive for DENV if a dilution neutralized 70% of the test virus at the following cut-off titers: 1:60 for DENV1 and DENV3, 1:80 for DENV2, and 1:40 for DENV4. A seroconversion was scored when the percent increase in reduction between a negative sample and a subsequent sample was greater than 2-fold. During the study period Iquitos experienced a DENV4 outbreak. Consequently, most new infections were presumed to be DENV4. The primary outcome of our trial was seroconversion over the course of the follow-up period.

#### Entomological surveys

To examine the impact of the ITCs on adult and immature *Ae*. *aegypti* abundance, longitudinal entomological surveillance was implemented at the beginning of the study, with a baseline entomological survey during October 2009. Larval and pupal surveys and adult mosquito collections using battery-operated aspirators [37] were conducted in all houses in treatment and control clusters. The first follow-up entomological survey occurred during January 2010 and subsequent follow-up surveys occurred during May 2010, February 2011 and May-June 2011. Either the CDC bottle bioassay [38] or WHO paper-based bioassay [39] were conducted to determine susceptibility of local *Ae*. *aegypti* populations to deltamethrin at baseline (Nov. 2009), May 2010, July 2010, February 2011, April 2011, and August 2011. Eggs were collected from clusters using ovitraps and were hatched and reared to adults (F0) for use in bioassays. Entomological data was a secondary outcome of the trial. The number of adult female *Ae*. *aegypti* per house had the greatest relevance to transmission risk [40, 41].

### Data analysis

Data were exported from a custom Microsoft Access database and analyzed using SAS statistical analysis software version 9.3 and R version 3.4.3. The effect of the intervention was estimated by calculating cluster-level summary measures and comparing them between arms by unpaired *t* test. For seroconversion, a rate per person-year was calculated for each cluster. Those at risk were those with a baseline PRNT measurement indicating they were not already positive for all four DENV serotypes. The numerator for the rate was the number of people who seroconverted to one or more serotypes between the surveys. The denominator was the person-time between the first and second surveys of those at risk. For the entomological endpoints, the area under the curve was calculated for each cluster. The follow-up values of the entomological endpoints were summarized in terms of the area under the curve (AUC) of the index against time estimated by trapezium rule, taking each time point as the mean survey date for each cluster [42, 43]. No subgroup or adjusted analyses were done.

## Results

At baseline, the demographic composition was similar between participants in intervention and control arms (Table 1).

**Table 1:** Baseline participant demographics.

	Intervention Arm	Control Arm
	Number (%) (n = 1721)	Number (%) (n = 1656)
Gender		
Male	739 (42.9%)	704 (42.5%)
Female	982 (57.1%)	952 (57.5%)
Age (years)		
3–20	772 (44.9%)	783 (47.3%)
21–40	584 (33.9%)	493 (29.8%)
> = 41	365 (21.2%)	380 (23.0%)
Mean	27.1	26.6

Seroprevalence and seroconversion data from individuals that provided samples at baseline and follow-up are presented in Table 2 (also see S1 Summary). In both the intervention and control arms, approximately 90% of participants had antibodies to at least one DENV serotype at baseline and approximately 85% of all participants were seronegative to at least one DENV serotype. There was a significant difference in overall seroconversion rates (seroconversion to any individual serotype or multiple serotypes; p<0.0001) between the intervention and control arms. The intervention arm had an average seroconversion rate of 50.6 per 100 person-years (PY) (CI: 29.9–71.9) and those in the control arm had an average seroconversion rate of 37.4 per 100 PY (CI: 15.2–51.7). This represents a statistically significant mean difference of 13.2 (CI: 12.0–14.4), with higher incidence in the intervention arm, or a difference equivalent to 35% of the average rate in the control arm.

**Table 2:** Seroprevalence at baseline and seroconversion in intervention (n = 918) and control (n = 1007) arms.

Cluster	Participants with baseline and follow-up samples (n)	Positive seroprevalence at baseline^a^ (n)	Participants at risk of seroconversion^b^ (n)	Seroconversion to different serotypes during the study (Seroconversion rate/100 person-years)^c^					
				DENV1 only	DENV2 only	DENV3 only	DENV4 only	Multiple serotypes^d^	Any serotype^e^^,^^f^
***Intervention arm***									
2	70	65 (92.9%)	66 (94.3%)	2.1	6.3	4.2	52.8	6.3	71.9
4	94	82 (87.2%)	89 (94.7%)	4.9	13.0	11.3	32.4	9.7	71.2
7	60	55 (91.7%)	55 (91.7%)	0.0	7.8	2.6	28.7	7.8	46.9
10	103	91 (88.3%)	91 (88.3%)	9.7	6.5	1.6	34.0	6.5	58.3
11	96	73 (76.0%)	73 (76.0%)	4.0	2.0	0.0	35.9	4.0	45.9
12	98	81 (82.7%)	81 (82.7%)	21.8	1.8	1.8	25.4	1.8	52.6
14	118	87 (73.7%)	87 (73.7%)	10.2	1.7	0.0	11.9	8.5	32.2
15	104	82 (78.8%)	82 (78.8%)	7.1	3.6	5.4	32.2	10.7	58.9
18	86	72 (83.7%)	72 (83.7%)	3.9	0.0	1.9	31.1	0.0	36.9
20	89	80 (89.9%)	80 (89.9%)	1.9	5.6	1.9	14.9	5.6	29.9
**Mean**	**91.8**	**82.8 (90.2%)**	**77.6 (84.5%)**	**7.0**	**4.9**	**3.1**	**29.4**	**6.2**	**50.6**^f^
Range	(60–118)	(55–108), (80.6%-98.8%)	(55–91), (73.7%-94.7%)	(0.0–21.8)	(0.0–13.0)	(0.0–11.3)	(11.9–53.8)	(0.0–10.7)	(29.9–71.9)
***Control arm***									
1	81	75 (92.6%)	65 (80.2%)	11.2	9.0	2.3	20.2	9.0	51.7
3	68	56 (82.4%)	58 (85.3%)	5.0	10.0	0.0	12.5	7.5	35.0
5	74	60 (81.1%)	69 (93.2%)	4.3	0.0	0.0	27.8	15.0	47.0
6	94	85 (90.4%)	80 (85.1%)	1.8	7.4	5.5	7.4	14.7	36.8
8	168	150 (89.3%)	143 (85.1%)	4.2	8.3	2.1	16.6	15.6	46.7
9	127	113 (89.0%)	119 (93.7%)	7.5	2.5	0.0	13.7	6.2	29.8
13	91	79 (86.8%)	74 (81.3%)	6.0	8.1	0.0	10.1	12.1	36.3
16	97	91 (93.8%)	82 (84.5%)	1.8	1.8	1.8	30.8	3.6	39.8
17	94	88 (93.6%)	75 (79.8%)	0.0	2.0	2.0	21.8	11.9	37.6
19	113	108 (95.6%)	88 (77.9%)	0.0	0.0	1.7	11.8	1.7	15.2
**Mean**	**100.7**	**93.7 (89.4%)**	**85.3 (84.7%)**	**4.2**	**4.8**	**1.6**	**17.0**	**9.9**	**37.4**^f^
Range	(68–168)	(58–160), (82.3%-95.7%)	(58–143), (77.9%-93.7%)	(0.0–11.2)	(0.0–10.0)	(0.0–5.5)	(7.4–30.8)	(1.7–15.6)	(15.2–51.7)

^a^The number of participants and total percentage of individuals with positive serological tests for 1 to 4 of the 4 DENV serotypes at baseline, among those with both baseline and follow-up samples

^b^The number of participants and total percentage of individuals at risk of seroconversion (i.e., without full immunity to all 4 DENV serotypes), among those with both baseline and follow-up samples

^c^DENV1-DENV4: Seroconversion to one, and only one, of these serotype during the course of the study

^d^Seroconversion to more than one of the four DENV serotypes during the course of the study

^e^Any seroconversion, including any single serotype conversion (DENV1-DENV4), or conversion to multiple serotypes, that took place during the study

^f^Any serotype conversion significant difference from the control arm; *t*-value -21.45, mean difference 13.2, 95% CI (-14.4, -12.0), p-value <0.0001

**Fig 3:**
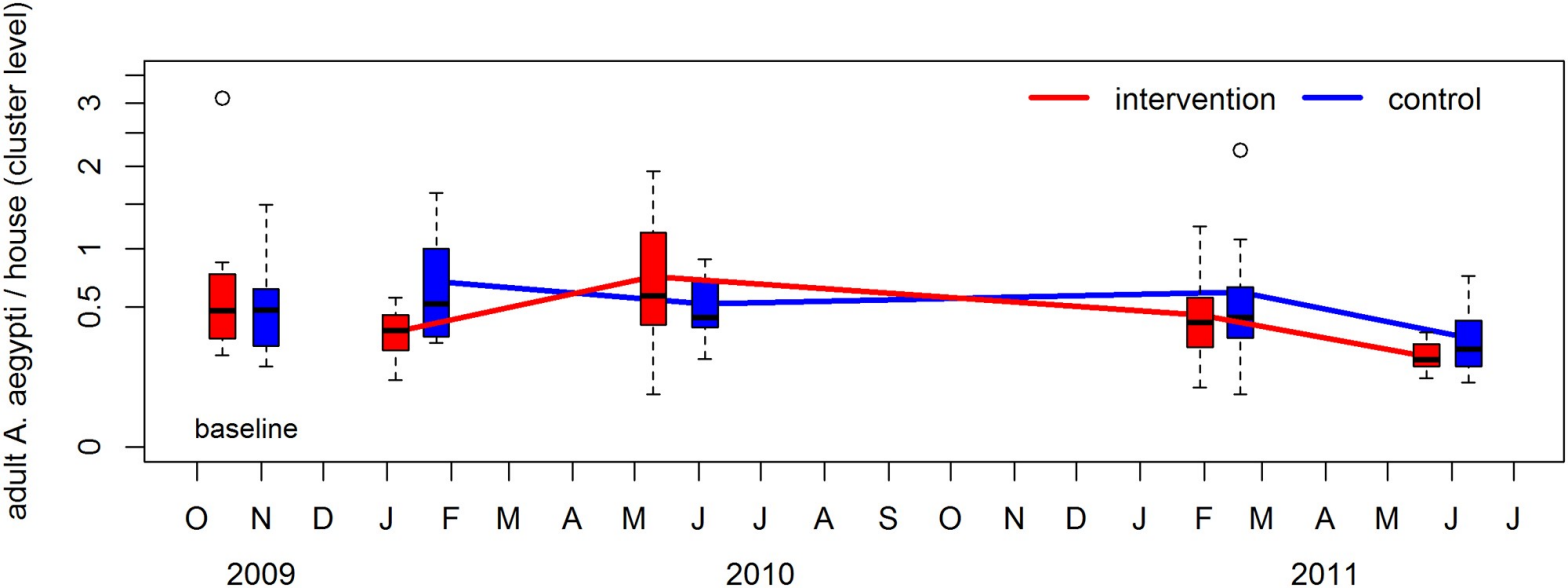
The average number of female *Ae*. *aegypti* collected per house. Intervention clusters are shown in red and control clusters are shown in blue. The upper and lower limits of each box are the interquartile range across clusters. Each ‘whisker’ (dashed line) extends to the most extreme data point which is no more than 1.5 times the interquartile range from the box. Circles represent values which are more extreme than the whiskers. The baseline data are represented by the boxes corresponding to October/November 2009.

**Fig 4:**
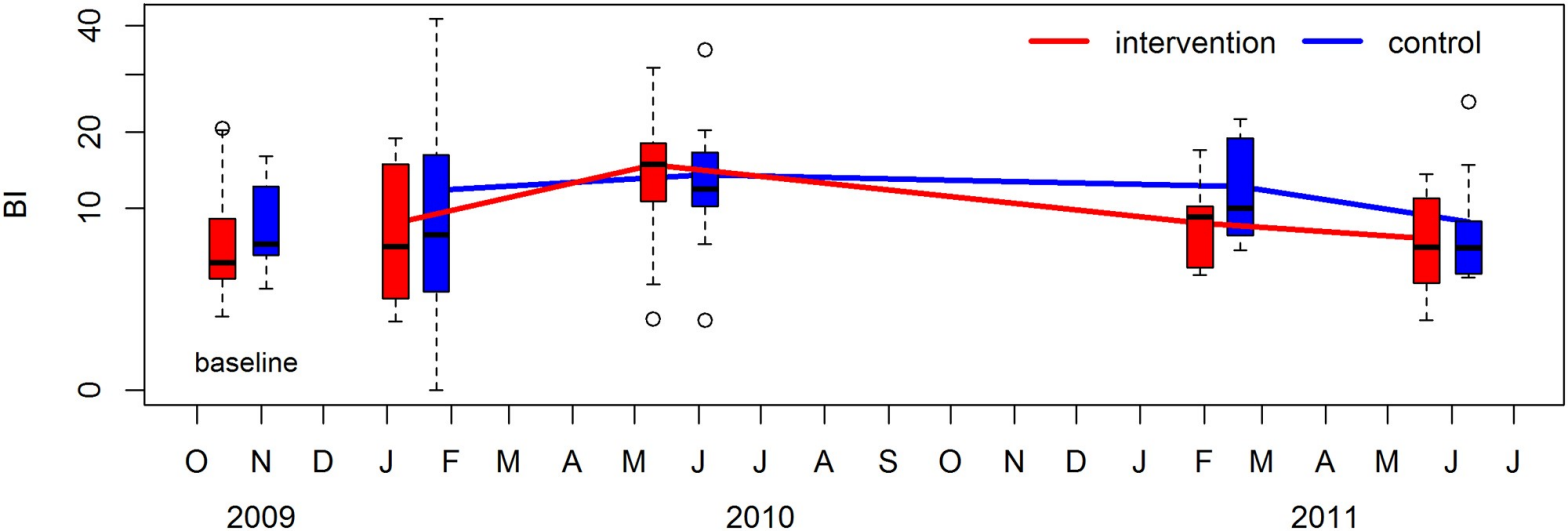
The Breteau Index, with results from intervention clusters shown in red and control clusters shown in blue. Boxes in the intervention period are joined by lines. The upper and lower limits of each box are the interquartile range across clusters. Each ‘whisker’ (dashed line) extends to the most extreme data point which is no more than 1.5 times the interquartile range from the box. Circles represent values which are more extreme than the whiskers. The baseline data are represented by the boxes corresponding to October/November 2009.

For entomological endpoints, the adult female *Aedes* index and the Breteau Index are shown in Figs 3 and 4; other indices are presented in supplementary material (S1 Summary). Overall, entomological indices were similar across treatment and control arms over the course of the study (Table 3). There were no significant differences detected between the intervention and control arms for any of the adult or immature *Ae*. *aegypti* indices that were measured.

**Table 3:** Summary of AUC analyses of entomological endpoints between intervention and control arms.

	Area under the curve (AUC): mean (SD) over clusters, based on time in days		Difference in AUC, intervention minus control (95% confidence interval) p-value^1^
	Intervention arm	Control arm	
Adult female *Aedes aegypti* per house	258 (134)	276 (135)	-17 (-144, 109) 0.77
Adult *Aedes aegypti* per house (males and females)	467 (230)	507 (219)	-42 (-253, 169) 0.68
Breteau Index	5444 (2343)	6286 (3014)	-841 (-3389, 1706) 0.50
Pupae per person	62.4 (32.5)	56.2 (35.1)	6.19 (-25.6, 38.0) 0.69
House Index	4112 (1500)	4868 (1721)	-756 (-2275, 764) 0.31
Container Index	1793 (842)	2123 (917)	-330 (-1157, 497) 0.41

^1^*t* test. Negative values favor the intervention.

Cone bioassays after re-treatment with KO-Tab 123 showed that the curtains did not immediately recover to the 100% bioefficacy observed at baseline. At 1 month following re-treatment (January 2011), average mortality for 36 ITCs was 74.5% (range: 48%-94%). By 9-months following re-treatment (August 2011) average mortality from 9 tested ITCs was 97.2% (range: 87.5%-100%).

Insecticide susceptibility data from CDC bottle bioassays demonstrated that the local *Ae*. *aegypti* population was fully susceptible to deltamethrin at baseline and remained fully susceptible when tested during May 2010 and July 2010. Resistance to deltamethrin was first detected during February 2011, when 24-hour mortality using the WHO bioassay dropped to 79.7% and was at a similar level (74.8%) during April 2011. Mortality fell further to 68.3% (using the CDC bottle bioassay) during August 2011.

## Discussion

The results indicate that participants living in the intervention arm were not better protected from DENV exposure than those in the control arm, despite the widespread use of ITCs. Although entomological indicators appeared to be lower in the intervention arm, most notably at the first follow-up survey, the differences were not statistically significant and were not sustained over the course of the study. These findings are in contrast with previous studies that have demonstrated clear entomological impacts of ITCs in cluster-randomized trials (CRT) [11, 13–16]. While previous studies of ITCs reported serological endpoints [11, 16], no other CRT had been powered based on seroconversion data. This trial was unique, therefore, because it benefited from multiple years of data collection from previous studies of dengue epidemiology in Iquitos. That stated, participants in the intervention arm were more likely to report reduced use of other mosquito products due to the feeling of protection from the ITCs compared to those in the control arm [17, 44].

That the participants in the intervention arm were more likely to seroconvert to DENV infection was surprising, especially given the high use of the ITCs [17, 44] and the promising entomological data reported in previous trials. Three factors, however, could have contributed to this unexpected result. First, despite being fabricated from a material that was expected to retain high insecticide levels over the course of several years, the ITCs quickly lost insecticidal efficacy, leading to the need for mass re-treatment only 1-year after they had been originally deployed. The 9-month serosurvey occurred before the ITCs were re-treated, which means that many were operating sub-optimally and could have potentially had reduced protective effectiveness. Second, a contributing factor may have been a false sense of security amongst the participants in the intervention arm; *i*.*e*., perhaps the presence of the highly visible ITCs created a belief in their protective power among the treated households, who subsequently did not employ any additional measures typically used to avoid exposure to mosquito bites. This possibility was suggested as an explanation for a similar outcome associated with the use of insecticide aerosols and mosquito coils in a meta-analysis of dengue interventions [45]. Indeed, comments by participants about the expected benefit of the ITCs were noted during focus group discussions held 6-months after the ITCs were deployed. Participants that had received ITCs commented that when the curtains were first hung, the household reduced their use of mosquito repellents and stopped fumigating because they felt that it was no longer necessary due to the presence of the ITCs [17]. Third, higher seroconversion rates in treatment than control clusters (Table 2) could reflect higher transmission risk for people in treatment areas; *i*.*e*., treatment and control clusters were not balanced for transmission risk.

Other complicating factors that should be considered for interpretation of our results are the close proximity of the study clusters, high mobility [46–49] of people in Iquitos, and study duration. Treated clusters were located across the street from untreated clusters. There were a few reports of family members in treated clusters loaning curtains to family members in an untreated cluster. The study population did not spend 100% of their time at their homes under protection of the ITCs. Theoretically, randomization would control for this, but a penalization for human movement patterns in and out of clusters was not included in our original sample size calculations or study design. After our CRT had been carried out, a series of publications offered recommendations for how to enhance the design CRTs to assess the epidemiological effects of interventions against *Aedes*-transmitted viruses [50–55]. The geographic spacing of clusters and accounting for human movement will be critical for future CRT study designs [53]. Of particular relevance to our study are insights that would minimize the complicating effects of movement and ways to gather movement data that will support quantifying a person’s time under coverage; e.g., see [53]. We measured seroconversions for only a single 9-month period (i.e., a single transmission season) and discontinued the study because of higher transmission rates observed in the ITC treated areas, which was associated with a high force of infection for DENV4 and unusually high entomological indices. A minimum of 2 transmission seasons is needed to account for interannual variation in virus transmission and vector population dynamics.

Results related to analyses of behaviors associated with ITC use provided the first indication that the ITCs were not functioning as expected. During focus group discussions conducted 6 months after the ITCs were hung, a common theme that emerged was the perception that the ITCs were working initially, but that their insecticidal impact seemed to wane rapidly. These observations were corroborated with quantitative data collected during a knowledge, attitudes, and practices (KAP) survey, which was conducted 9-months after ITCs were deployed. A third of the KAP survey respondents reported that they observed a temporary drop in the amount of mosquitoes in their homes. Overall, the surveyed population perceived that mosquito numbers were only lowered for an average of 3.3 months after the ITCs were hung [17].

Bioassays detected an increase in resistance to deltamethrin in the local *Ae*. *aegypti* population over the course of the study. Resistance was first detected in early 2011, after ITCs had been deployed for over a year, soon after they were re-treated with deltamethrin. This initial detection of resistance was worrying, because it could indicate that low concentrations of insecticide on ITCs that were not adequately loaded with deltamethrin (the reason why the re-treatment was carried out) had already begun to select for deltamethrin resistance in the local mosquito population.

While the outcomes of our study were unanticipated, they highlight several key challenges related to the widespread community use of ITCs to reduce dengue transmission. First, key dynamics influencing the potential of this tool in Iquitos appeared to have been dependent on human behavior in ways other than those we had considered. For example, while the community readily adopted and used the ITCs [44], they may have done so at the expense of other protective measures. Hence households using ITCs that were later confirmed to be faulty, were at greater risk of seroconverting to dengue. Future ITC-based interventions will need to take great care in emphasizing that ITCs should supplement, rather than replace, existing protective strategies. Second, the quality of the insecticide-treated material is fundamental to the success of the intervention. Failures in efficacy can lead to difficulty in interpreting results from a trial. The reduced insecticidal effect of the ITCs was associated with the initial detection of deltamethrin resistance in the local *Ae*. *aegypti* population. This is particularly troubling in the case of *Ae*. *aegypti* because arbovirus control programs are heavily reliant on a limited number of approved insecticides. All insecticide-based interventions should include rigorous quality control during mass production of the finished trial product, to minimize the possibility that sublethal doses of insecticide are deployed in target localities, which can lead to multiple negative consequences.

Since this trial was completed a growing body of evidence indicates that the potential for ITCs as vector control tools for reducing DENV transmission likely depends more on how effectively they act as physical barriers to prevent mosquito ingress, than on how well they deliver and sustain insecticidal efficacy. ITCs tightly fitted as screens to windows (and doors) reduced indoor mosquito densities for long periods, even when they were untreated or after the insecticide treatment had been lost [56, 57]. Although this is good news from the perspective of insecticide resistance, screening windows and doors will not be possible at every location. Where communities live in houses with numerous openings to maximize air movement (*e*.*g*. high eaves, floor to ceiling doorways, etc.), as in Iquitos, Thailand [43] and numerous other locations, such screening would be impossible without major changes in home construction. Identifying effective means of protecting those communities against dengue and the other infections transmitted by *Ae*. *aegypti* remains an obstinate challenge that will require integration of multiple strategies, including approaches that are intersectoral and go beyond traditional methods for vector control [58].

## Supporting information

S1 Protocol Trial Protocol. (PDF)

S1 Checklist CONSORT checklist. (DOC)

S1 Summary Summary entomological indices. (CSV)
