# Supplementary material for: The impact of insecticide treated curtains on dengue virus transmission: A cluster randomized trial in Iquitos, Peru
Source: PLoS Negl Trop Dis. 2020 Apr 10;14(4):e0008097. doi: 10.1371/journal.pntd.0008097 (PMC7176142; doi:10.1371/journal.pntd.0008097)
Supplement: S1 Protocol — (PDF) [file pntd.0008097.s001.pdf]

PART II: APPLICATION SUMMARY INFORMATION

A. **Protocol Number**: NMRCD.2009.0007

B. **Protocol Title**: Can insecticide-treated curtains prevent transmission of dengue?

C. **Relevant Work Unit Title and Work Unit Number**: 6000 RAD1.S.B0302

D. **Principal Investigators**:

Phillip McCall, Ph.D., Vector Group, Liverpool School of Tropical Medicine, Pembroke Place, Liverpool L3 5QA, UK, Tel: +44 151 705 3132, Fax: +44 151 705 3369, Email: mccall@liv.ac.uk

Crystyan Siles, MD, VEID, NAMRU-6, Iquitos, Peru, Tel: 51-065-581732, Email: crystyansiles@gmail.com.

E. **Submitting Investigator/IRB Certification Number**:
Crystyan Siles, M.D., Citi training: 04/07/2016

F. **Co-Investigators**:

Amy Morrison, Ph.D., Project Scientist II: Director, Iquitos Field Laboratory, NAMRU-6-Iquitos, Department of Entomology and NAMRU-6-Peru, University of California, Davis, One Shields Ave., Davis, CA 95616. IRB Certification Number: 0004, Email: amy.aegypti@gmail.com. Citi training: 11/13/2014

Audrey Lenhart, Ph.D., MPH, Senior Research Associate, Liverpool School of Tropical Medicine, Vector Group, Pembroke Place, Liverpool L3 5QA, UK. Email: alenhart@liv.ac.uk. Citi training: 01/16/2015

Moises Sihuincha, M.D., Director, Department of Internal Medicine, Hospital de Apoyo Iquitos. IRB Certification Number: 0601109105. Email: Sihuincha@hotmail.com. Citi training: 05/30/2013

Neal Alexander, Ph.D., Statistician, London School of Hygiene and Tropical Medicine, Keppel Street, London WC1E 7HT, UK. Email: neal.alexander@lshtm.ac.uk. Citi training: 06/09/09

Valerie Paz Soldan, Ph.D., Health Office for Latin America, School of Public Health & Tropical Medicine, Tulane University, Calle Pedro Martinto #184, Barranco – Lima 4, Peru. Email: vpazsold@tulane.edu. Citi training: 06/10/2013

Thomas Scott, Ph.D., Department of Entomology, University of California, Davis, One Shields Ave., Davis, CA 95616. Email: twscott@ucdavis.edu. Citi training: 08/18/2013

John Elder, Ph.D., Division of Health Promotion & Behavioral Science, School of Public Health, San Diego State University, 9245 Sky Park Court, #221, San Diego, CA 92123. Email: jelder@mail.sdsu.edu. Citi training: 06/11/2014

G. **Research Locations**:

Human subjects, epidemiological and entomological surveillance: Iquitos, Peru; Serology: NAMRU-6-Peru, Data analysis: Iquitos, Peru, NAMRU-6-Peru, Liverpool School of Tropical Medicine, UK, London School of Hygiene and Tropical Medicine, UK.

H. **Lead Agency**: Liverpool School of Tropical Medicine

I. **Collaborating Domestic Institution(s):**

Naval Medical Research Unit No.6, Iquitos and Lima, Peru

Department of Entomology, University of California, Davis, Davis, CA, 95616

Tulane University, New Orleans, LA 70118

San Diego State University, San Diego, CA 92123

J. **International Approval Agency:** DISA Loreto

K. **Proposed Start and End Dates**: July 2009 – December 2016

L. **Projected/Estimated Total number of Enrollees**: A maximum of 2000 households will be enrolled for the entomology and KAP surveys. A maximum of 4,000 individuals resident in these households will be enrolled for the serosurvey.

M. **Inclusions**:
- Persons aged 3 years or older with parental permission
- Adults consenting to participate in the study living in the study area
- Assent to participation and a parentally signed ICD for 8-17 year old persons

N. **Exclusions**:
- Persons younger than 3 years old
- Temporary visitors to the study areas
- Adults who do not consent to participate
- 8-17 year old persons who do not assent to participate in the study or who do not have parental permission to participate in the study

O. **Anticipated Risks**: The risks of drawing blood from a vein include discomfort at the site of puncture, possible bruising and swelling around the puncture site, and uncommonly, faintness from the procedure, and rarely, an infection. Risks from insecticide exposure are minimal and all products have WHOPES approval and are labeled for their intended use.

P. **Proposed Risk Reduction Methodologies and Provisions**: Only trained personnel will perform venipuncture procedures using sterile, single use needles, alcohol/betadine wipes and bandages.

Q. **Protocol Abstract**:

This study is designed to demonstrate the efficacy of insecticide treated materials (ITMs) in reducing dengue virus transmission and *Ae. aegypti* intra-domiciliary and peri-domestic abundance in intervention groups compared to control groups. A cluster-randomized controlled trial will be conducted over 30 months to measure whether ITMs can reduce dengue virus transmission and dengue vector activity in treated households and communities. Efficacy of the intervention will be determined by measuring dengue virus seroconversion rates and

a range of entomological indices. Acceptance and sustainability of ITMs will be evaluated using socio-behavioral longitudinal surveys and focus group discussions.

PART III: PROTOCOL CRITICAL ELEMENTS

A. SCIENTIFIC BACKGROUND AND OBJECTIVES

Purpose: Dengue fever (DF) and the potentially lethal forms, dengue hemorrhagic fever/ dengue shock syndrome (DHF/ DSS) are rapidly growing public health problems worldwide, with an estimated 50 million infections and at least 24,000 deaths (mainly among children) annually (WHO 2002). In the absence of a vaccine, control of the peridomestic vector mosquito, *Aedes aegypti* (and to a lesser extent, *Aedes albopictus*), is the only available preventive measure. Existing control methods target immature mosquito stages, requiring continuous effort by communities (Parks and Lloyd 2004), and are difficult to sustain (Nathan and Knudsen 1991) and/ or expensive. Moreover, by targeting only developing stages, control impacts only on vector density and does not directly reduce dengue transmission. A more effective and appropriate community-level intervention is urgently needed. Recently, we showed that insecticide-treated materials (ITMs) can reduce dengue vector populations (Kroeger *et al.* 2006; Lenhart *et al.* 2008). Community based control of adult dengue vector mosquitoes is now possible. This work comprises a definitive trial of ITMs to measure their effect on virus transmission, and comprehensively assess suitability of ITMs as household-level interventions, by addressing two questions:

1. Can insecticide-treated curtains reduce dengue transmission in treated households (individual or household effect) and in treated communities (community or mass effect)?
2. What human behavioral factors influence adoption, maintenance and dissemination of this technology among householders?

Study Format: The study is designed to demonstrate the efficacy of a simple intervention in reducing dengue virus transmission and *Ae. aegypti* intra-domiciliary and peri-domestic abundance in intervention groups compared to control groups. A cluster-randomized controlled trial will be conducted over 30 months to measure whether ITMs can reduce dengue virus transmission and dengue vector activity in treated households and communities. Efficacy of the intervention will be determined by measuring dengue virus seroconversion rates and a range of entomological indices. Acceptance and sustainability of ITMs will be evaluated using socio-behavioral longitudinal surveys and focus group discussions.

Specific Aims:

- Determine if insecticide-treated curtains reduce dengue transmission in treated households (individual or household effect) and in treated communities (community or mass effect)
- Investigate the human behavioral factors that influence adoption, maintenance and dissemination of ITMs among householders

B. EXPERIMENTAL METHODS

1. Experimental Procedures and Rationale including information to show that studies in animals or *in vitro* systems could not address the hypothesis(es) under test.

Research Methods: The study will be conducted in the city of Iquitos, in north-eastern Peru. The study is subdivided into 3 components: (1) assessing the effect of ITMs on dengue virus transmission, (2) assessing the effect of ITMs on household vector infestation and breeding, and (3) studying the factors that influence how and why people use the ITMs.

Study Area and Design: The study will be carried out in the district of San Juan (see appendix 2 from original Wellcome grant for detailed description), as no dengue research or intervention trials have been or are currently being undertaken there. In the last 10 years, this area has undergone extensive population growth, and dengue transmission rates have increased. According to local Ministry of Health data, *Ae. aegypti* indices in San Juan are now similar to those in the most highly infested areas in Iquitos.

This is a cluster-randomized controlled trial, in which geographically defined clusters will be randomized to either receive ITMs or to act as controls (no ITMs). All households in control clusters will be offered ITMs at the end of the study. Clusters will comprise on average 2 city blocks, with a minimum of 70 households per cluster. The study will be conducted in 20 clusters (10 treatment and 10 control clusters), with an estimated 460 people per cluster. Our sample size calculation is based on existing data from Iquitos and elaborated in section 2 below (also see appendix 1 from original Wellcome grant).

Procedures:

Blood Sample Collection:

Venipuncture or fingerstick procedures will be performed using standard aseptic techniques. An experienced phlebotomist (or study physician) will take the blood sample from an antecubital vein. Three ml (0.61 teaspoons) of whole venous blood will be obtained from each volunteer. The blood will be collected in one Vacutainer® collection tube (red top) without anticoagulant. If venipuncture is unacceptable to a study subject, the finger prick method will be used for collection of a person's first sample. Providing each participant with a choice of methods will increase compliance and reduce the drop-out rate during subsequent collections. For finger pricks, we will use a BD genie safety lancet and microtainer tube system. After cleaning a finger with 70% alcohol, the sterile lancet will be used to the puncture skin. The finger will be squeezed by the phlebotomist forming a large drop of blood which is held over the microtainer tube. Capillary action draws the blood into the 1 ml tubes. Sera will be separated by centrifugation at 2500 rpm for 10 minutes at 4^0C, transferred to cryo-vials and stored at -20^0C.

Laboratory Procedures:

<u>Plaque Reduction Neutralization Test (PRNT):</u> A modified protocol of Morens *et al.* (1985) will be followed. Test sera will be diluted two fold in media (EMEM + Pen./Strep.) from 1:40 to 1:640. Two hundred ul media containing 40 to 80 PFU of assay virus will be mixed with 200 ul diluted test serum and then incubated at 4^0 C for 15 hours. In triplicate, 100 ul virus-serum mixture will be added to 0.5 ml media containing 1.5 x 10^5 BHK21 cells and then added to a well of a 24 well tissue culture plate and incubated at 37^0 C with 5% CO_2 for 3 hrs. The cells will then be overlaid with 0.5 ml of overlay media (0.6% Carboxymethyl Cellulose, MEM w/o Phenol Red, 10% FBS, 0.075% $NaHCO_3$ and Pen./Strep.) and incubated at 37^0 C with 5% CO_2 for 5 days. The media will be removed, and the cells rinsed with H_2O and stained with 0.5 ml/well stain solution (0.1% (w/v) Naphthol Blue Black, 1.36% (w/v) Sodium Acetate, and 6% (v/v) Glacial Acetic Acid) for 30 min. The stain will be removed and the plaques will be counted. The results will be expressed as the serum dilution, determined by probit analysis, that reduced the number of plaques by 70% compared to that of normal human serum at the same dilution**.**

Entomological Surveys:

Longitudinal entomological surveillance will be implemented at the beginning of the study and carried out at 6 month intervals for the duration of the study. The primary objective of the entomological evaluation in this study is to determine if the adult and larval mosquito population densities are affected by the ITMs. This will consist of:

<u>Larval and Pupal Collections:</u> At each house examined, we will inspect all water-holding containers located indoors and outdoors, following methods described by Getis *et al.* (2003) and Morrison *et al.* (2004 a & b). We will classify each container by type (e.g., water storage tank, barrel, plastic container, tire, flower pot), measure its volumetric capacity, and score it for solar exposure (0, total shade; 1, total sun), lid status (yes, no), water management (how the container is filled with water, usefulness to resident), location in relationship to vegetation (potential nutrition source) and presence of larvicide. A standard data sheet for recording this information will be used. Currently in Iquitos, we have an experienced team of 12 collectors and 2 supervisors that survey between 60-100 houses during a 6 hr workday, or an average of 1,800 houses per month. In our previous Iquitos studies the time taken for sampling averaged 9 minutes per house (range 2-170 minutes) and varied with the number of adult mosquitoes and containers detected with immature *Aedes*.

Pupae and larvae will be collected from water holding containers with fine mesh nets or eye droppers. In large containers, water will be agitated in a circular motion and pupae and larvae drawn into a vortex where they are easily collected (Tun-Lin *et al.* 1995; A. Morrison & T.W. Scott, unpublished data). Pupae will be transferred to 120 ml whirl pack plastic bags (Nasco, Fort Atkinson, WI), labeled with the houses and container code, and transported to the field laboratory where they will be counted, and placed in plastic emergence vials (#3.5, #9 Dram, Thornton Plastics, Salt Lake City, UT), maximum

30 per vial. Each subsequent day, emerged adults will be identified to species and counted by sex. To maintain essential quality control, we have found that having survey teams work in a group and having an entomological supervisor accompanying the team (10 collectors) greatly improves quality of the surveys. In addition, a 2nd supervisor will carry out spot checks of randomly selected subsamples of houses examined earlier that day (Soper 1967). Data sheets will be reviewed daily to identify possible errors and inconsistencies in data collection. Immediate feedback reinforces collector's skills and reminds them of the importance of accuracy in their jobs.

Adult Captures: Adult *Ae. aegypti* will be collected with CDC backpack aspirators (Scott *et al.* 1993 a & b);. The end of each aspirator tube is fitted with a 120 ml plastic urine collection cup. After aspirating the house, the collector will cap, remove, and label the cup and then store it until transport to the laboratory the same morning. At the field laboratory mosquitoes will be sedated by cold (4°C), identified, and counted.

Household Questionnaires: Prior to performing entomological and serological surveys, we will administer a household questionnaire to the head of the household. Information will be obtained on the number, age and sex of occupants, dimensions of the property, housing materials, method of cooking, water use patterns, type of sewage disposal, and insecticide use. The number of occupants and dimensions of the property are essential to calculate mosquito indices using persons or hectares in the denominator. The remaining information will be analyzed for dengue risk factors.

Calculation of Density Indices: With the information generated from entomological surveys and household questionnaires we will calculate the following indices of *Ae. aegypti* density: house index (HI, % of houses infested with immature *Ae. aegypti*), container index (CI, % wet containers positive for *Ae. aegypti* larvae or pupae), Breteau index (BI, No. of containers positive for immature *Ae. aegypti*/100 households surveyed), household pupal index (HPI = % houses infested with *Ae. aegypti* pupae), pupae per household (Pu/Hse = No. pupae collected/No. households surveyed), pupae per person (Pu/per = No. pupae collected/No. people living in households surveyed), pupae per hectare (Pu/Ha = No. pupae collected/No. hectares surveyed [sum of lots surveyed]), adult index (AI = % houses infested with adult *Ae. aegypti*), adults per household (AA/Hse = No. adult *Ae. aegypti* collected/No. households surveyed), adults per person (AA/per = No. adult *Ae. aegypti* collected/No. people living in households surveyed), adults per hectare (AA/Ha = No. adult *Ae. aegypti* collected/No. hectares surveyed [sum of lots surveys]), adult female index (AFI = % houses infested with adult female *Ae. aegypti*), adult females per household (AAF/Hse = No. adult *Ae. aegypti* females collected/No. households surveyed), adult females per person (AAF/per = No. adult *Ae. aegypti* females collected/No. people living in households surveyed), and adult females per hectare (AAF/Ha = No. adult *Ae. aegypti* females collected/No. hectares surveyed).

Behavior Study:

The primary objective of the human behavioral component is to determine the factors associated with adoption and continued use of ITMs, including the most effective diffusion mechanism (channel of communication) for ITM promotion. The study design consists of a combination of focus groups and socio-behavioral (KAP) surveys to be implemented at three different stages of the study.

Focus group discussions: At baseline, ten focus group discussions will be conducted to assess attitudes towards ITMs, perspectives on issues that may affect acceptance, correct usage and eventual adoption of ITMs, and to discuss potential channels of communication for ITM promotion. This will provide preliminary information to inform construction of questions for the socio-behavioral (KAP) surveys regarding ITM adoption (i.e. regular, correct use of a valued item).

At the time of the second and third serological surveys, 10 focus group discussions will again be conducted to obtain more in-depth information about adopters' and non-adopters' perspectives on the ITMs (e.g. what they liked/or not; why they used ITMs/or not; what might compel those who did not use ITMs to reconsider; why ITM use was not maintained). Purposive sampling will be used for all three series of focus group discussions, although criteria for selection of participants into the focus groups will vary: in the first series, groups will be split by gender; the second series by adopters vs. non-adopters of ITCs (identified by observing homes for evidence of adoption) and by gender; in the third series, groups will be split based on whether individuals are continuing ITC users or not, and by gender. In each of the series of focus groups, there will be two additional focus groups gathering information from key informants – one focus group with community leaders, and one focus group with health professionals from that community. All focus groups will be conducted by a social scientist experienced at conducting such discussions.

Socio-behavioral surveys: This study has two components: 1) an observation of appropriate ITM use and maintenance behavior, and 2) a survey on knowledge, attitudes and practices (KAP) regarding ITMs, diffusion of information regarding ITMs, as well as socio-demographic information.

1. Observation of ITM use: In health behavior research, the gold measurement standard involves some objective observation of the behavior. A questionnaire for the direct observation of ITM use will be developed and field tested. This questionnaire will be applied at all households along with the survey (see below for timing).
2. KAP survey: Baseline data regarding people's knowledge, attitudes and current practices associated with ITM use will be collected via a survey in intervention and control households, for an estimated maximum sample size of 2000 individuals. To ensure representation of both males and females, at each house we will sample the adult whose birthday is next. A subsequent survey will be administered to the same individuals in these households 9 months later (to coincide with the serological survey) and will include questions related to appropriate ITM adoption and maintenance, as well as channels of communication for diffusion of information about ITMs. A final survey will be implemented to coincide with the 27-month post-intervention serological survey.

Frequencies and means of the main variables of interest from the observation questionnaire and survey will be estimated, and multivariate logistic regressions conducted to examine associations between socio-demographic and behavioral data and the main outcomes: ITM adoption and maintenance.

ITM distribution
After the baseline surveys are completed, the ITMs will be distributed to the clusters that were randomly selected to receive them. The ITMs will be delivered to each household and hung by the members of the study team according to the preferences of the householder. The team hanging the ITMs will keep a logbook in which they record the quantity and locations of the ITMs hung in each participating house.

Re-treatment of the ITMs. Prior to the re-treatment of the ITMs, the KAP survey team will visit all households to deliver a new information sheet which explains the need for the curtains to be re-treated. In addition to providing the information sheet, the team will also explain the situation verbally to the householders and answer any questions they may have. The local study team, together with representatives from the DISA's vector control unit in Iquitos, will be trained in how to re-treat the curtains with deltamethrin using KO Tab 123 (Bayer) per the manufacturer's guidelines. On a block by block basis, all ITMs will be collected and taken to a central facility where they will be washed and re-treated and allowed to dry. Our bioassay data indicate that the amount of insecticide remaining on the curtains is too low to have any appreciable effect on mosquitoes, so it shouldn't be necessary to remove whatever small levels of insecticide remain prior to re-treatment. Even if small residual levels of insecticide remain on the curtains, there should not be any increased risk to human handlers after the re-application of the insecticide, as the levels of insecticide would still be far below the thresholds of human toxicity. Deltamethrin has been extensively evaluated by the WHO Pesticide Evaluation Scheme (WHOPES), and is considered one of the safest insecticides for use on insecticide treated materials precisely because of its low mammalian toxicity.
They will be returned to householders within 2 days and re-hung by members of the field team. Curtains will be labeled according to household and location within the household to ensure that after treatment they are returned to their original location.

Ideally, the re-treatment will commence in mid-October 2010 and should be complete by the end of November 2010. This should ensure that the insecticidal efficacy of all curtains has been restored prior to the peak dengue transmission season (Dec.-April). Routine monitoring of insecticidal efficacy will remain in place to detect any future failures and respond to them in a timely manner.

2. **A sample Size Determination with Statistical Power Calculation (if indicated), including the total number of volunteers to be enrolled in the entire study and in any specific groups included within the study, whether they are military or civilian, male or female, and the age range of volunteers.**

Based on the most recently available data from previous dengue cohort studies in Iquitos (see appendix 1 from original Wellcome grant), we expect baseline seroprevalence rates to exceed 80%. Our sample size calculation is based on

unpublished data from 2 studies using PRNT status at 9-month intervals (NAMRU-6 standard protocol) in residents of Iquitos. In the first study (2004-5), the last two serosurveys showed average rates of seroconversion (i.e. adding of at least one serotype) of 0.29/year and 0.25/year; between-cluster coefficients of variation were 0.37 and 0.18, and numbers of people at risk of seroconversion were 90 and 153, respectively. We used Hayes & Bennett's (1999) sample size method for binary data with the following parameters, which are chosen to lie within the range of values found in the previous studies: average of 120 people at risk per cluster; between-cluster coefficient of variation of 0.30; significance level 5% (two-sided); seroconversion rate in control and intervention clusters of 0.25 and 0.1375/year respectively (55% efficacy). With these parameters, 10 clusters per arm are needed for 90% power. This will provide 4000 blood samples at baseline (2000 per arm, more than the 1350 required to detect a difference) and an estimated 2000 at each subsequent sample round, assuming that >50% of the population remains susceptible to >1 serotype. In a later study (2006-7), the between cluster variation was less (k = 0.2), although seroconversion remained high, at 0.25/year. Using these parameters, a sample size of 7 clusters/arm would detect a 50% reduction in seroconversion. However, we will use the more conservative estimate of 10 clusters/arm, as this gives greater sensitivity to detect changes in seroconversion at high between-cluster variation rates.

3. Procedures that will be performed by other than NAVMEDRSCHCEN institutions (if any)

- Subject enrollment and ITM distribution will be carried out by Liverpool School of Tropical Medicine team located at 456 Avenida Fitzcarrald, Iquitos, Peru.
- Specimen collection, and specimen preparation and focus group discussion will be carried out at NAMRU-6-Iquitos.
- Entomological surveys be processed and managed at the UC-Davis Field Laboratory in Iquitos, Peru (377 Casa Callao)
- PRNT will be done at (or by personnel at) NAMRU-6-Lima.
- Data analysis will be carried out at Liverpool School of Tropical Medicine.
- Re-treatment of the ITMs will be carried out by the Liverpool School of Tropical Medicine team together with collaborators from the DISA-Iquitos

4. Detailed Inclusion and Exclusion Criteria. Exclusions must include justification.

For individuals providing blood samples at 9-month intervals for serology, the inclusion and exclusion criteria are:

Inclusion:

- Persons aged 3 years or older with parental permission
- Adults consenting to participate in the study living in the study area
- Assent to participation and a parentally signed ICD for 8-17 year old persons

Exclusion:

- Persons younger than 3 years old
- Temporary visitors to the study areas
- Adults who do not consent to participate
- 8-17 year old persons who do not assent to participate in the study or who do not have parental permission to participate in the study

5. Required Equipment and Supplies (as needed to ensure proper coordination of research effort)

This study is fully funded by the Wellcome Trust and all participating laboratories have the required equipment and consumable supplies to do the study.

C. ORGANIZATION OF RESEARCH EFFORT (RESEARCH PLAN)

1. **Duties and Responsibilities of Investigators and other individuals involved in the protocol. Delineate responsibilities for ethical review, administrative oversight etc as needed.**

Dr. Philip McCall **(Principal Investigator)** and Dr. Audrey Lenhart from the Liverpool School of Tropical Medicine have overseen multiple field trials of ITMs for dengue vector control in Latin America and SE Asia over the past 7 years. Dr. McCall is the PI of this study and Dr Lenhart is the co-investigator responsible for coordinating all aspects of the research.

Dr. Crystyan Siles (**NAMRU-6 lead investigator**) is a study physician in the Virology and Emerging Infections Department in Iquitos. He will be responsible for laboratory analysis, data management, quality control, and manuscript preparation for this study.

Dr. Neal Alexander has experience of design, conduct and analysis of cluster-randomized trials against vector-borne diseases including dengue, filariasis and malaria. He is the co-investigator on the project with responsibility for design and analysis.

Dr. Valerie Paz Soldan is a Peruvian-American social scientist with experience studying human behavior and disease patterns in Iquitos. She is the co-investigator responsible for the coordination and analysis of behavioral research activities.

Prof. Thomas Scott and Dr. Amy Morrison have over 10 years experience in researching dengue vectors and epidemiology in Iquitos. They are co-investigators who will act in advisory roles as well as in coordination of entomological activities.

Dr. Moises Sihuincha if the Chief of the Tropical Medicine and Infectious Diseases Department at Hospital Apoyo Iquitos. He is the co-investigator responsible for the coordination of project efforts with local and national Peruvian officials.

Prof. John Elder is a social scientist with experience studying human behavior patterns in Iquitos. He is a scientific advisor to the study.

2. **Multicenter organizational plan with responsibilities for IRB review and approval.**

IRB approval is required from the Liverpool School of Tropical Medicine, London School of Tropical Medicine and Hygiene in the UK, Tulane University and University of California, Davis, USA, and the Naval Medical Research Unit No.6, Lima, Peru which will serve as the local Peruvian IRB. At present Inter-IRB agreements with Tulane University and UC Davis are being negotiated to defer review to the NAMRU-6 IRB.

D. RISKS AND DISCOMFORTS TO RESEARCH VOLUNTEERS

1. **List of the significant risks to the Volunteer and the safeguards in place to minimize risk and deal with emergencies.**

Although risk associated with venipuncture is minimal, we will reduce risk of pain and infection to a minimum by using highly skilled staff with over 3-10 years of experience working on similar projects, universal precautions, and will have continuing education courses for our phlebotomists. We have developed 4 week certificate courses on biosafety (includes universal precautions, blood drawing technique, and patient interaction), and ethics (emphasis on the informed consent process). All participating staff will be required to complete these courses with annual renewal. The information from these studies is not expected to be sensitive or lead to any negative ramifications such as legal or employment risks, nor is there is a possibility of physical, psychological, social or legal injury from participation in this study and standard measures to protect confidentiality should be sufficient.

2. **A description of how appropriate anonymity will be maintained for any human samples or identifiable data collected or used.**

The information from these studies is not expected to be sensitive or lead to any negative ramifications such as legal or employment risks, and standard measures to protect confidentiality should be sufficient. All paper data forms will be stored in locked files or cabinets in the LSTM Iquitos field office or NAMRU-6 Lima in a specified storage facility with limited access. Computer data files are password protected to allow access only to appropriate study personnel. All files sent to consultants, collaborators and co-investigators will have names and addresses removed. Household codes that are georeferenced in our GIS for the city will provide x,y coordinates for spatial statistical analysis, which will be linked to entomological and serological data at these sites. No participant identifiers, beyond the participant's code will be included within our GIS or shown on printed maps or in publications of the study area. Databases generated from these studies will only contain code identifiers and never names. As stated above, it will be clearly explained to participants that information from these studies will be kept strictly confidential.

Data will be used only to address study objectives. All data from the study will be shared with the MOH personnel to support existing control and surveillance activities (e.g. entomological indices we provide will complement MOH survey data so that vector control activities can be targeted to appropriate locations), as well as help shape future control strategies in Iquitos. It is anticipated that the results of the study will be presented to the Peruvian Health Authorities, the scientific community via oral presentations, and written reports and publications in scientific journals.

3. **Special Risks to Pregnant or Potentially Pregnant Women Volunteers**

NONE

4. **Safety Precautions and Emergency Procedures**

There is no unusual risk for those participating in this study. There is no unusual risk to those conducting this research. Individuals who participate will benefit from application of insecticide treated curtains. At the end of the study, participants from the non-treated area will be offered the same treated curtains as the treated-areas. Insecticide treated mosquito nets have been deployed around Iquitos as part of the national control program and are likely to result in a decrease of mosquito biting activity. Adverse toxicological effects are not likely since we will be following label instructions. The risks from the insecticide treated curtains are considered minimal; some people may experience coughing, sneezing, eye irritation and may also have some skin sensitivity to the insecticide in the first 24 hours after removal from commercial packaging and with prolonged direct contact. This is likely to be only mild and temporary. The curtains will be deployed by trained field teams after they are placed in an open shaded place for > 24 hr and a pamphlet with describing their proper use provided to the study participants.

Venipunture will be performed by qualified phlebotomists Universal standard safety precautions including aseptic technique and single-use sterile needles will be followed when handling blood samples. No significant adverse effects are expected from the phlebotomy. Occasional bruising and a slight risk of infection at the site of the venipuncture are the most serious injuries that a volunteer can receive from participating in the study.

5. **Assessment of Sufficiency of Plans to Deal With Untoward Events or Injuries**

Serious and unexpected adverse experiences will be immediately reported by study physicians to local health care workers, and Dr. Lenhart or Dr. Sihuincha at the LSTM Iquitos field office, located at 456 Avenida Fitzcarrald, Iquitos and reported to the PI, Dr. Philip McCall. Dr. Lenhart and/or Dr. McCall will inform LSTM, NAMRU-6, and the Loreto Regional Health Department by telephone or e-mail within 24 hours of notification for Severe Adverse Events. Appropriate reports will then be submitted within required time frames.

6. **Qualification of Medical Monitor and Medical Support Personnel**

A waiver for the medical monitor is requested for this study. All study physicians are licensed to practice medicine in Iquitos. Only study physicians or trained phlebotomists will collect specimens.

E. DESCRIPTION OF THE SYSTEM FOR MAINTENANCE OF RECORDS

1. Experimental Data

The information from these studies is not expected to be sensitive or lead to any negative ramifications such as legal or employment risks, and standard measures to protect confidentiality should be sufficient. All paper data forms will be stored in locked files or cabinets in the LSTM Iquitos field office or NAMRU-6 Lima in a specified storage facility with limited access. Access to computer data files will be password protected to allow exclusive access to appropriate study personnel. All files sent to consultants, collaborators and co-investigators will have names and addresses removed. Household codes that are georeferenced in our GIS for the city will provide x, y coordinates for spatial statistical analysis, which will be linked to entomological and serological data at these sites. No participant identifiers, beyond the participant's code will be included within our GIS or shown on printed maps or in publications of the study area.

2. Research Protocol, Consent Forms, and Related Documents for Protection of Human Research Volunteers.

All official protocol files (protocol, IRB minutes, and approvals) will be maintained at the NAMRU-6 under password protection. All consent forms and questionnaires will be maintained in the LSTM Iquitos field office or NAMRU-6 Iquitos Laboratory, UC Davis Iquitos Field Laboratory or NAMRU-6 Lima Facility and stored under limited access.

3. Individual Medical Records: Not Applicable

PART IV: REQUIRED ENCLOSURES

1. **Approved application to the Wellcome Trust, with Appendices**
2. **Reviewer's comments on Wellcome Trust grant application**
3. **PermaNet MSDS**
4. **PermaNet curtains manufacturer's informational booklet**

REFERENCES

Getis, A., *et al*. (2003) Characteristics of the spatial pattern of the dengue vector, *Aedes aegypti*, in Iquitos, Peru. *Am J Trop Med Hyg*, 69(5): 494-505.

Hayes & Bennett S (1999) Simple sample size calculation for cluster-randomized trials. *Int. J Epid*. 28 319-26.

Kroeger A, Lenhart A, Ochoa M, Villegas E, Levy M, Alexander N & McCall PJ. (2006) Effective control of dengue vectors with curtains and water container covers treated with insecticide in Mexico and Venezuela: cluster randomised trials. *BMJ*, 332, 1247-52.

Lenhart A, Orelus N, Maskill R, Alexander N, Streit T & McCall PJ. (2008) Insecticide treated bednets to control dengue vectors: preliminary evidence from a controlled trial in Haiti. *Trop. Med. Int. Health*, 13(1), 56-67.

Morens DM, Halstead SB, Repik PM, Putvatana R, Raybourne N. (1985) Simplified plaque reduction neutralization assay for dengue viruses by Semimicro Methods in BHK-21 cells: Comparison of BHK suspension test with Standard Plaque Reduction Neutralization. *J. Clin. Microbiol*. 22, 250-4.

Morrison, A.C., *et al*. (2004a) Temporal and geographic patterns of *Aedes aegypti* (Diptera: Culicidae) production in Iquitos, Peru. *J Med Entomol*, 41(6): p. 1123-42.

Morrison, A.C., *et al*. (2004b) Evaluation of a sampling methodology for rapid assessment of *Aedes aegypti* infestation levels in Iquitos, Peru. *J Med Entomol*, 41(3): p. 502-10.

Nathan M & Knudsen A. (1991) *Ae. aegypti* infestation characteristics in several Caribbean countries and implications for integrated community-based control. *J. Am. Mosq. Con. Assoc.* 7, 400-4.

Parks W & Lloyd L. (2004) Planning social mobilization and communication for dengue fever prevention and control. Geneva: WHO.

Scott, T.W., *et al*. (1993a) Blood-feeding patterns of *Aedes aegypti* (Diptera: Culicidae) collected in a rural Thai village. *J Med Entomol*, 30(5): 922-7.

Scott, T., *et al*. (1993b) Detection of multiple blood feeding in *Aedes aegypti* (Diptera: Culicidae) during a single gonotrophic cycle using a histologic technique. *J Med Entomol*, 30: 94-99.

Soper 1967.

Tun-Lin W, Maung-Maung-Mya, Sein-Maung-Than, Tin-Maung-Maung (1995). Rapid and efficient removal of immature *Aedes aegypti* in metal drums by sweep net and modified sweeping method. *Southeast Asian J Trop Med Public Health*. 26(4):754-9.

WHO. (2002) Dengue and Dengue Haemorrhagic Fever. Fact Sheet 117. Geneva.
